# Five-year outcomes of single-anastomosis duodeno–ileal bypass with sleeve gastrectomy (SADI-S) in adults with type 1 diabetes mellitus

**DOI:** 10.1007/s12020-026-04664-x

**Published:** 2026-06-01

**Authors:** Ravi S. Rao, Gabrielle Hogan, Aditya Rao, Ashlen Sweeny, Munish Mehta

**Affiliations:** 1 Perth Surgical & Bariatrics, Perth, WA Australia; 2 The Royal Australasian College of Surgeons, Melbourne, VIC Australia; 3 Royal Perth Hospital, Perth, WA Australia; 4 Alstom, Perth, WA Australia; 5 BigData Scientists Pty Ltd, Perth, Australia

**Keywords:** Metabolic surgery, SADI-S, type 1 diabetes, glycemic variability, continuous glucose monitoring, bariatric outcomes

## Abstract

**Background:**

Managing obesity in adults with type 1 diabetes mellitus (T1DM) is challenging because insulin dependence often coexists with significant insulin resistance. While metabolic surgery is well established in type 2 diabetes, long-term data in T1DM remain limited. This study evaluates five-year outcomes of single-anastomosis duodeno–ileal bypass with sleeve gastrectomy (SADI-S) in adults with T1DM.

**Materials and methods:**

Adults with T1DM and obesity who underwent primary SADI-S at a single center were followed for up to five years. Outcomes included weight, BMI, HbA1c, total daily insulin requirements, continuous glucose monitoring (CGM) metrics, nutritional status, peri-operative safety, and patient-reported quality-of-life measures. CGM data were analyzed for time in range, glycemic variability, time above range, and time below range. Patient-reported outcomes included the SF-12, Diabetes Treatment Satisfaction Questionnaire, and BAROS.

**Results:**

Eight patients contributed five-year outcome data. Mean BMI decreased from 38.6 kg/m² to 26.4 kg/m². HbA1c improved from 8.4% to 7.1%, and total daily insulin requirements decreased by approximately 45%. CGM data demonstrated increased time in range, reduced glycemic variability, and stable rates of hypoglycemia. Quality-of-life scores improved across all validated domains. Two cases of early postoperative euglycemic diabetic ketoacidosis occurred before implementation of extended inpatient monitoring; none occurred thereafter. No patient developed protein-calorie malnutrition or severe micronutrient deficiency.

**Conclusions:**

SADI-S produced sustained weight loss, improved glycemic stability, reduced insulin requirements, and acceptable nutritional outcomes over five years in adults with T1DM. These findings support SADI-S as a physiologically favorable metabolic procedure in carefully selected insulin-treated individuals.

## Introduction

Type 1 diabetes mellitus (T1DM) is characterized by autoimmune destruction of pancreatic β-cells leading to lifelong absolute insulin deficiency [1]. Persistent hyperglycemia contributes to microvascular and macrovascular complications despite advances in insulin formulations, delivery technology, and real-time continuous glucose monitoring (CGM) [2–4]. Increasingly, many adults with T1DM present with concomitant obesity, visceral adiposity, insulin resistance, and metabolic features resembling type 2 diabetes—an overlap often described as “double diabetes” [3, 5]. This combination substantially increases insulin requirements, glycemic variability, and cardiometabolic risk.

Although metabolic surgery is well established for type 2 diabetes, evidence in T1DM remains limited and heterogeneous, with published data primarily derived from small observational cohorts and case series across a range of procedures [6–10]. Studies of Roux-en-Y gastric bypass (RYGB) and sleeve gastrectomy (SG) have consistently shown substantial weight loss and reductions in insulin dose, but improvements in HbA1c and glycemic stability remain modest and inconsistent [8, 11–14]. Furthermore, RYGB is associated with rapid gastric emptying, exaggerated incretin responses, early postprandial hyperglycemia, and late reactive hypoglycemia, contributing to glycemic instability in individuals relying on exogenous insulin [14–17]. CGM studies demonstrate widened glycemic excursions and unpredictable insulin requirements after RYGB in T1DM [14, 15].

Single-anastomosis duodeno–ileal bypass with sleeve gastrectomy (SADI-S) is an evolution of the biliopancreatic diversion–duodenal switch, designed to maintain metabolic effectiveness while reducing malabsorption [9, 18–20]. Our group has previously reported favorable nutritional and metabolic outcomes of SADI-S in broader clinical populations, including Australian cohorts [10, 21, 22]. By preserving the pylorus, moderating foregut exclusion, and providing controlled distal intestinal stimulation, SADI-S may offer a more physiologically stable glycemic profile than RYGB or one-anastomosis gastric bypass in adults with T1DM. Therefore, the primary aim of this study was to evaluate the five-year efficacy and safety of SADI-S in adults with T1DM, specifically assessing weight loss, glycemic control, insulin requirements, and continuous glucose monitoring (CGM) metrics.

## Methods

### Study design and ethics 

This was a retrospective analysis of a prospectively maintained database of adults with T1DM who underwent SADI-S between January 2017 and December 2023. Ethics approval was not required because only de-identified clinical data were analyzed, consistent with the National Statement on Ethical Conduct in Human Research (2007; updated 2018), Paragraph 5.1.22, Page 79 [23]. All patients provided written consent for anonymous use of clinical information for research and publication. All surgeries were performed by a single senior advanced laparoscopic and bariatric surgeon.

### Participants 

Twelve adults with confirmed T1DM were enrolled to undergo SADI-S during the study period. All participants were expected to complete follow-up assessments including weight, HbA1c, insulin requirements, CGM, nutritional intake, and patient-reported outcome measures. All patients had a specialist-confirmed diagnosis of T1DM based on clinical history and documentation from endocrinologists or diabetes nurse educators. Available C-peptide values were consistent with absolute insulin deficiency, supporting differentiation from latent autoimmune diabetes in adults or insulin-requiring type 2 diabetes. Preoperative evaluation included assessment of smoking status, renal function, liver health, and nutritional parameters. Use of medications known to affect glycemic control or perioperative risk, including sodium-glucose cotransporter-2 (SGLT2) inhibitors, was reviewed. All patients were instructed to use CGM both before and after surgery. Insulin delivery method (multiple daily injections or insulin pump therapy with or without automation) was recorded at baseline.

### Surgical technique 

All procedures were performed laparoscopically using a standardized technique previously described. A sleeve gastrectomy was fashioned over a 36-Fr bougie. The duodenum was divided approximately 3 cm distal to the pylorus. A single end-to-side duodenoileostomy was created 300 cm proximal to the ileocecal valve and closed with a running 2 − 0 Polysorb suture using the EndoStitch device. The 300-cm common channel length reflected prior work demonstrating balanced metabolic efficacy and nutritional safety.

### Postoperative care and follow-up

Initially, a postoperative protocol involving day-1 discharge was applied, following standard practice for non-T1DM patients. After early cases of euglycemic diabetic ketoacidosis (EuDKA), the protocol was revised to mandate a minimum 3-day inpatient stay for all subsequent patients. Postoperatively, all patients were prescribed a standardized daily nutritional supplementation protocol, consistent with our previously published regimen for SADI-S cohorts [21]. This specific daily regimen included thiamine (12 mg), methylcobalamin (500 µg), folate (800 µg), vitamin A acetate (10,000 IU; modified to beta-carotene for women planning pregnancy), and vitamin E succinate (20 IU). Additionally, patients received vitamin K1 (1 mg), vitamin D3 (3,000 IU), iron picolinate (18 mg), zinc picolinate (18 mg), copper gluconate (2 mg), and pyridoxine (5 mg). Follow-up visits were scheduled at 2 weeks, 3 months, 6 months, 12 months, 18 months, 24 months, and annually thereafter.

Biochemical and Nutritional Monitoring Monitoring included serum vitamins A, D, E, K, B12, folate, iron studies, calcium, parathyroid hormone, albumin, international normalized ratio (INR), and renal function. These were recorded at 6, 12, 18, and 24 months, and annually thereafter.

Continuous Glucose Monitoring CGM data were obtained retrospectively from routine clinical care. Patients used either the Freestyle Libre 2 system or a Dexcom device. Each collected dataset represented a 14-day recording period obtained preoperatively and at postoperative intervals. CGM metrics included mean glucose, glucose management indicator (GMI), time in range (TIR; 3.9–10 mmol/L), coefficient of variation (CV), time above range (TAR), and time below range (TBR). Four patients contributed datasets for ≥ 24 months, and two provided full five-year profiles.

Patient-Reported Outcomes PROMs included the SF-12, Diabetes Treatment Satisfaction Questionnaire (DTSQ), Bariatric Analysis and Reporting Outcome System (BAROS), and a procedure-specific quality-of-life instrument assessing hunger, satiety, energy, and diabetes self-management. PROMs were obtained preoperatively and annually. Higher scores indicated greater satisfaction or better quality of life across all scales.

### Statistical analysis

Given the small cohort and incomplete long-term datasets, analyses were primarily descriptive. Due to the retrospective, observational nature of this study and the clinical rarity of the cohort, a formal a priori sample size calculation was not feasible. Continuous variables are reported as mean ± SD, and categorical variables as n (%). Where paired values were available, exploratory paired Wilcoxon signed-rank tests were undertaken without adjustment for multiple comparisons; a non-parametric approach was selected given the small sample size [24]. Missing data were not imputed. CGM trajectories were interpreted descriptively in accordance with international consensus guidelines.

## Results

Twelve adults with T1DM underwent SADI-S between 2017 and 2023. Three relocated overseas and were lost to follow-up. Of the remaining nine, eight contributed complete postoperative datasets, while one provided baseline data only. The final analyzable cohort consisted of eight patients (six females, two males) with a mean age of 41 ± 9 years and a mean preoperative BMI of 38.6 ± 5.2 kg/m². The mean duration of diabetes was 19 ± 7 years, and at baseline, the cohort included three patients utilizing automated insulin pumps and five utilizing multiple daily injections. Overall follow-up for primary outcomes was 75% (9/12). Four patients contributed longitudinal CGM datasets extending ≥ 24 months, and two had complete five-year profiles. None of the twelve patients were active smokers. No patient used SGLT2 inhibitors, and none had preoperative renal impairment or hypoalbuminemia. Three patients had ultrasonographic or biochemical evidence of non-alcoholic fatty liver disease. All patients used CGM both pre- and postoperatively.

**Fig. 1:**
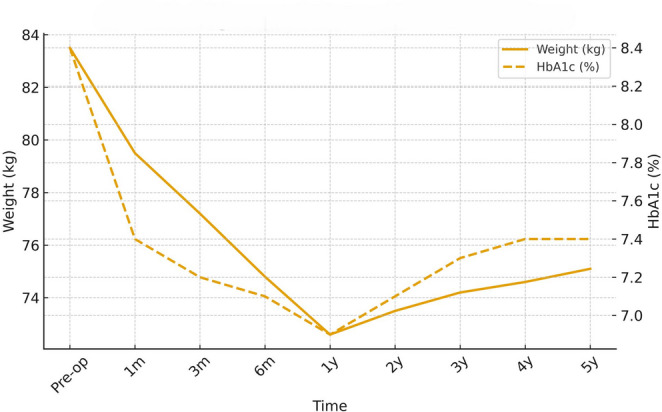
Weight and HbA1c over time following SADI-S. Abbreviations: HbA1c, glycated hemoglobin; SADI-S, single-anastomosis duodeno–ileal bypass with sleeve gastrectomy. Line graphs demonstrating mean weight (kg) and HbA1c (%) across the 60-month follow-up period

Weight, BMI, and Glycemic Outcomes SADI-S produced durable and progressive weight loss. Mean weight declined from 108 ± 12 kg at baseline to 73 ± 7 kg at 60 months (approximately 30–34% total weight loss) (Table 1). BMI decreased from 38.6 ± 5.2 kg/m² to 26.4 ± 3.4 kg/m² at five years. HbA1c improved from 8.4 ± 1.1% to 7.1–7.3% across long-term follow-up (Table 1) (Fig. 1). Although modest, the improvement was durable and accompanied by reductions in glycemic variability and insulin requirements.

**Table 1:** Longitudinal Glycemic and Anthropometric Outcomes

Outcome	Baseline	12 m	24 m	36 m	48 m	60 m
Weight (kg)	108 ± 12	79 ± 10	76 ± 8	74 ± 7	73 ± 7	73 ± 7
BMI (kg/m²)	38.6 ± 5.2	28.3 ± 4.1	27.4 ± 3.8	26.7 ± 3.5	26.5 ± 3.4	26.4 ± 3.4
% TWL	NA	30 ± 4	32 ± 5	33 ± 5	34 ± 5	34 ± 5
HbA1c (%)	8.4 ± 1.1	7.3 ± 0.6	7.2 ± 0.5	7.2 ± 0.5	7.1 ± 0.5	7.1 ± 0.5
Total daily insulin (U)	42 ± 5	26 ± 4	25 ± 4	24 ± 4	24 ± 4	23 ± 4

Abbreviations: M, months; BMI, body mass index; %TWL, percentage total weight loss; NA, not applicable; HbA1c, hemoglobin A1c

### Insulin requirements 

**Fig. 2:**
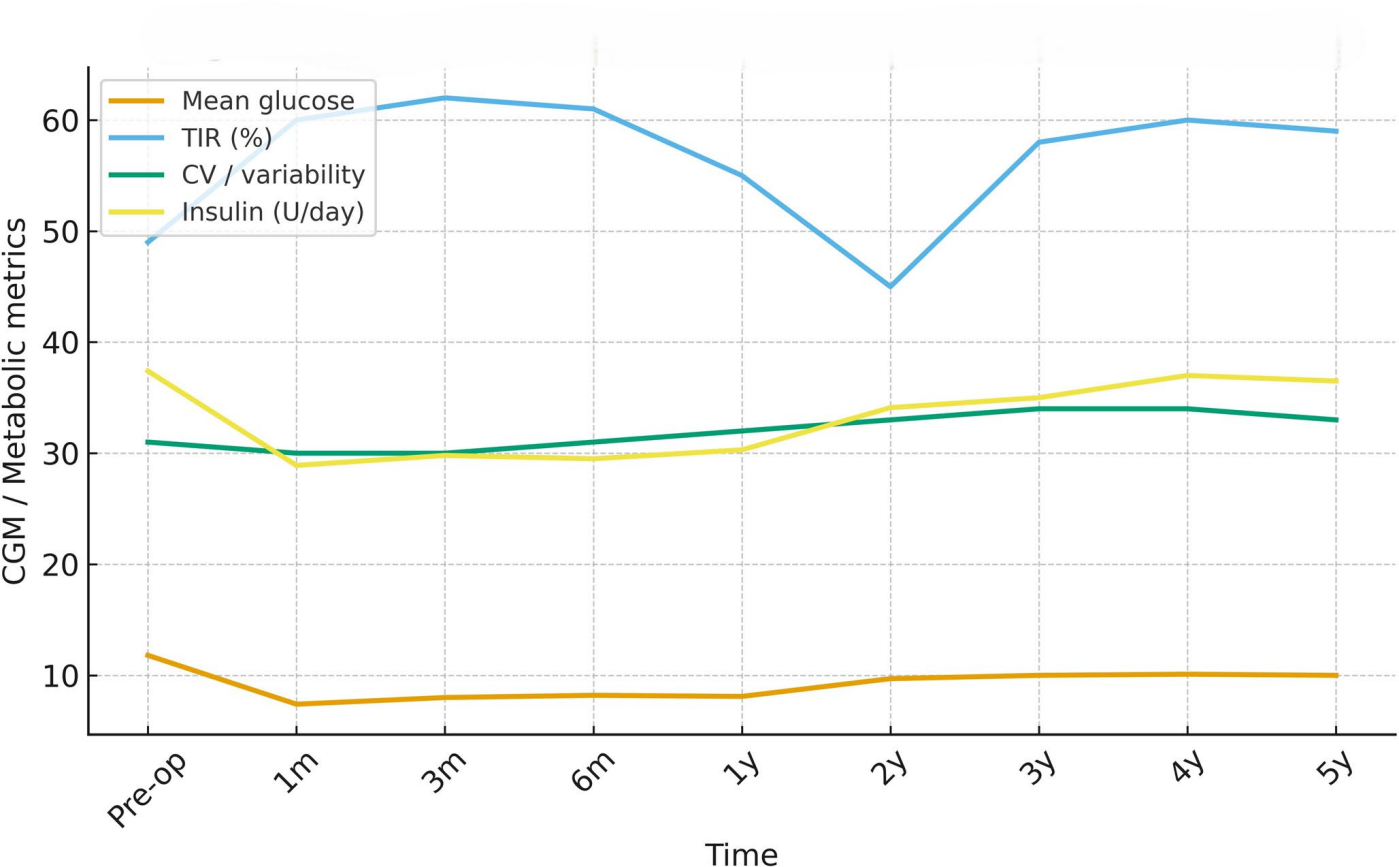
Composite continuous glucose monitoring (CGM) metrics after SADI-S. Abbreviations: CGM, continuous glucose monitoring; TIR, time in range; TAR, time above range; TBR, time below range; GMI, glucose management indicator; CV, coefficient of variation. Longitudinal CGM-derived metrics including time in range (TIR), time above range (TAR), time below range (TBR), mean glucose, glucose management indicator (GMI), and coefficient of variation (CV) from baseline to 60 months

**Fig. 3:**
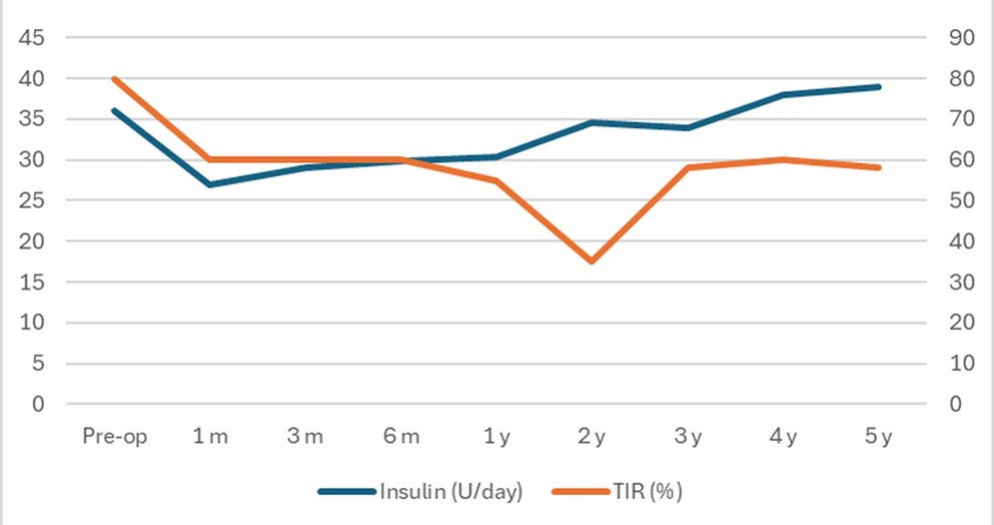
Insulin dose and time-in-range trends following SADI-S in adults with type 1 diabetes mellitus. Abbreviations: TIR, time in range. Longitudinal changes in total daily insulin requirements and continuous glucose monitoring. (CGM)–derived time-in-range (70–180 mg/dL) are displayed from pre-operative baseline to 5-year follow-up

Total daily insulin use declined from 42 ± 5 units/day at baseline to 23–28 units/day at follow-up, representing an approximate 45% reduction (Table 1) (Figs. 2 and 3). No patient discontinued insulin therapy.

Continuous Glucose Monitoring Outcomes TIR improved from approximately 45% preoperatively to 60–68% during months 24–60 (Table 2). Two patients with complete five-year datasets demonstrated stable TIR of 68 ± 5% at 60 months. Mean glucose and GMI improved progressively, while CV approached the recommended target of < 36% (Table 2) (Figs. 2 and 3). Time above range (TAR) and time below range (TBR) both improved progressively, reflecting reduced hyper- and hypoglycemia burden (Table 2). CGM profiles showed fewer large meal-related spikes and reduced “saw-tooth” oscillations compared with patterns frequently reported after RYGB in T1DM, which are characterized by early postprandial hyperglycemia and late reactive hypoglycemia. These CGM findings reflect the four patients with longitudinal datasets; 48- and 60-month values represent the two patients with full profiles.

**Table 2:** Longitudinal Continuous Glucose Monitoring Metrics

CGM Metric	Baseline	12 m	24 m	36 m	48 m	60 m
TIR (%; 3.9–10 mmol/L)	45 ± 8	58 ± 7	63 ± 6	64 ± 6	66 ± 5	68 ± 5
Mean glucose (mmol/L)	10.8 ± 1.2	9.2 ± 0.9	8.8 ± 0.8	8.7 ± 0.8	8.6 ± 0.7	8.5 ± 0.7
GMI (%)	8.5 ± 0.4	7.6 ± 0.3	7.4 ± 0.3	7.3 ± 0.3	7.3 ± 0.3	7.2 ± 0.3
CV (%)	40 ± 5	36 ± 4	34 ± 4	33 ± 4	32 ± 4	32 ± 3
TAR (%)	48 ± 10	36 ± 8	32 ± 7	30 ± 7	29 ± 6	28 ± 6
TBR (%)	7 ± 3	6 ± 2	5 ± 2	5 ± 2	5 ± 2	4 ± 2

Abbreviations: CGM, continuous glucose monitoring; TIR, time in range; GMI, glucose management indicator; CV, coefficient of variation; TAR, time above range; TBR, time below range

**Fig. 4:**
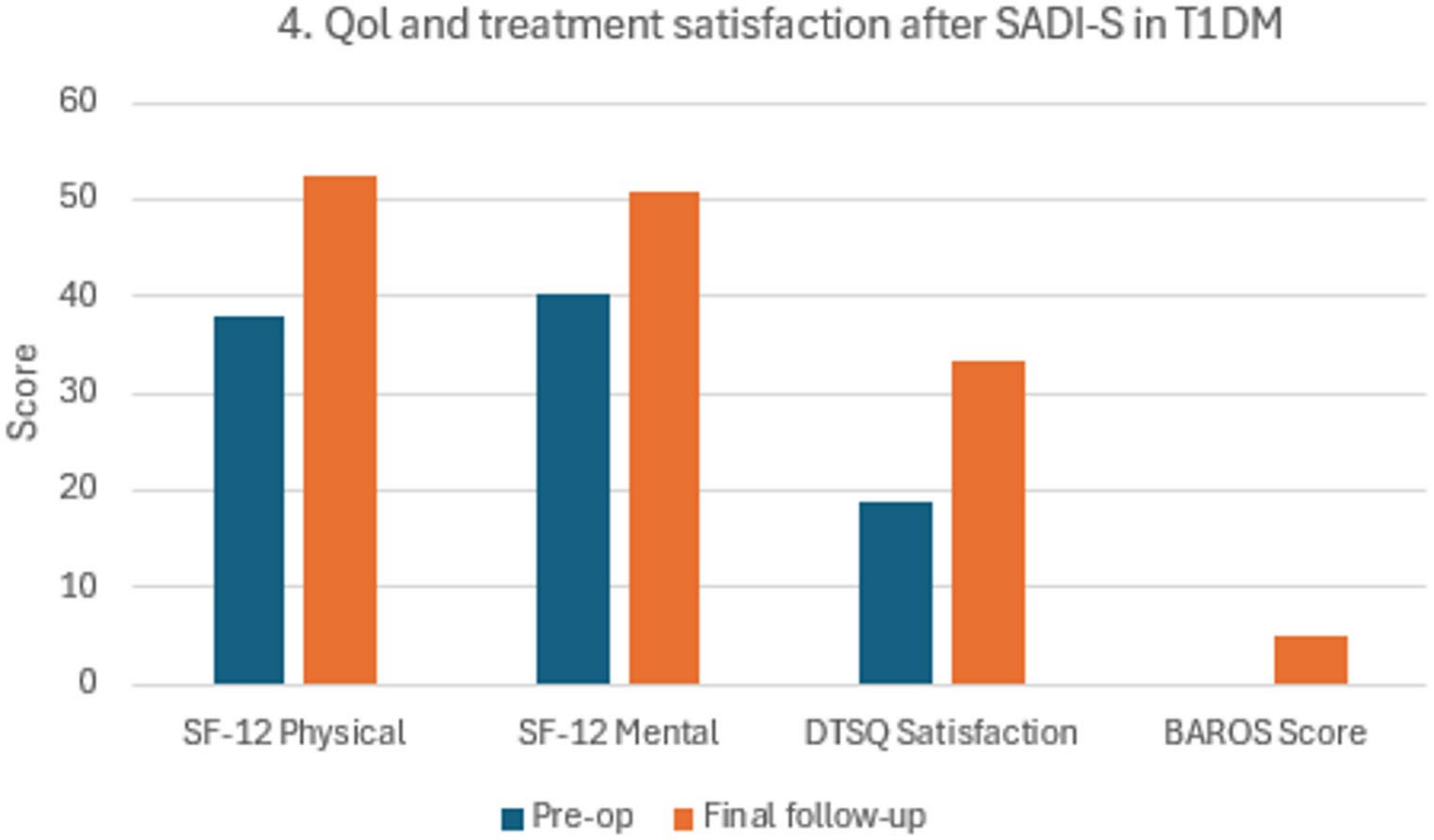
Quality-of-life and treatment satisfaction after SADI-S. Abbreviations: SF-12, 12-item Short-Form Health Survey; DTSQ, Diabetes Treatment Satisfaction Questionnaire; BAROS, Bariatric Analysis and Reporting Outcome System; QoL, quality of life; SADI-S, single-anastomosis duodeno–ileal bypass with sleeve gastrectomy. Trends in SF-12 physical and mental component scores, Diabetes Treatment Satisfaction Questionnaire (DTSQ) satisfaction scores, and BAROS categories over 60 months after SADI-S

Patient-Reported Outcomes PROMs were completed by eight patients. SF-12 physical and mental scores improved and remained stable through five years. DTSQ scores indicated higher treatment satisfaction and reductions in perceived hyper- and hypoglycemia. BAROS scores ranged from very good to excellent (Table 3). Patients reported improved energy, reduced hunger, and enhanced diabetes self-management (Fig. 4).

**Table 3:** Patient-Reported Outcomes

Measure	Baseline	12 m	24 m	36 m	48 m	60 m
SF-12 PCS	38 ± 6	48 ± 5	50 ± 4	50 ± 4	51 ± 4	52 ± 4
SF-12 MCS	42 ± 7	50 ± 6	52 ± 5	52 ± 5	53 ± 5	53 ± 5
DTSQ satisfaction	22 ± 4	32 ± 3	33 ± 3	33 ± 3	34 ± 3	34 ± 3
DTSQ hyperglycemia	4.5 ± 1.2	2.1 ± 0.9	2.0 ± 0.8	2.0 ± 0.8	1.9 ± 0.7	1.8 ± 0.7
DTSQ hypoglycemia	3.9 ± 1.0	2.0 ± 0.8	1.9 ± 0.8	1.9 ± 0.8	1.8 ± 0.7	1.8 ± 0.7
BAROS total	—	4.8 ± 0.6	5.0 ± 0.5	5.1 ± 0.5	5.1 ± 0.5	5.2 ± 0.5
Procedure-specific QoL composite	52 ± 10	78 ± 8	80 ± 7	82 ± 7	82 ± 7	83 ± 6

Abbreviations: SF-12 PCS, 12-Item Short Form Survey – Physical Component Summary; SF-12 MCS, 12-Item Short Form Survey – Mental Component Summary; DTSQ, Diabetes Treatment Satisfaction Questionnaire; BAROS, Bariatric Analysis and Reporting Outcome System; QoL, quality of life

Nutritional Outcomes Micronutrient deficiencies were infrequent and correctable. Nutritional parameters remained largely stable at the five-year follow-up, with mean cohort values demonstrating adequate overall nutritional status: serum albumin (40.0 ± 1.3 g/L), corrected calcium (2.25 ± 0.06 mmol/L), ferritin (149 ± 82 µg/L), hemoglobin (143.8 ± 8.1 g/L), and 25-OH Vitamin D (63.6 ± 22.7 nmol/L). Serum albumin remained within normal limits for all patients. Vitamin D deficiency occurred in two patients; combined vitamin A and calcium deficiency in one; and combined vitamin A, folate, and mild vitamin D deficiency at five years in one.

### Complications 

Two cases of EuDKA occurred early in the series, both following day-1 discharge. After protocol modification to a minimum 3-day inpatient stay, no further EuDKA occurred. No patient experienced internal hernia, stricture, marginal ulceration, bile reflux, severe hypoglycemia requiring assistance, reoperation, or mortality.

## Discussion

The present study provides, to our knowledge, the first five-year analysis of SADI-S in adults with T1DM. The results demonstrate sustained weight reduction, moderate but durable HbA1c improvement, approximately 45% reduction in insulin requirements, enhanced glycemic stability, and broad improvements in patient-reported quality of life. These findings extend the emerging role of metabolic surgery in T1DM and underscore several mechanisms by which SADI-S may confer physiological advantages in this group.

The management of obesity in adults with T1DM presents distinct metabolic challenges. Absolute insulin deficiency requires lifelong replacement, yet many patients also demonstrate significant insulin resistance associated with excess adiposity, a phenotype often described as double diabetes [3, 5]. This dual burden complicates weight loss, glycemic stability, and long-term cardiometabolic outcomes despite advances in insulin delivery systems and CGM. Evidence supporting bariatric or metabolic surgery in T1DM has predominantly focused on RYGB and SG. These procedures consistently reduce weight and insulin requirements, but improvements in HbA1c and glycemic variability remain modest and unpredictable. In particular, RYGB has been associated with accelerated gastric emptying, rapid postprandial glucose rises, reactive hypoglycemia, and EuDKA, highlighting unique safety considerations in this population [14–16, 25, 26].

Metabolic surgery improves glycemic control in T2DM through mechanisms including enhanced glucagon-like peptide-1 and peptide YY secretion, foregut exclusion, bile-acid signaling, intestinal gluconeogenesis, microbiome changes, and substantial weight-related improvements in insulin sensitivity. Although β-cell failure prevents remission in T1DM, several of these mechanisms remain clinically relevant. In this study, marked reductions in visceral adiposity likely contributed to improved insulin sensitivity, while reductions in glycemic variability allowed safer and more predictable insulin titration. TIR, GMI, and CV support the concept that metabolic surgery can meaningfully enhance glycemic stability in T1DM [27, 28].

A key observation in this cohort was the improvement in CGM-derived glycemic stability. TIR increased from approximately 45% to 60–68% across five years, while CV approached consensus targets (< 36%). These findings contrast with published RYGB data in T1DM, where rapid gastric emptying, exaggerated incretin responses, and foregut bypass frequently produce early hyperglycemia followed by late hypoglycemia [14–17].

Several characteristics of SADI-S may help explain the more physiological glycemic response observed. Preservation of the pylorus enables controlled and predictable gastric emptying, thereby reducing rapid carbohydrate delivery to the distal small intestine and blunting the exaggerated peaks and troughs seen following RYGB and, to a lesser extent, one-anastomosis gastric bypass (OAGB). The single-loop diversion maintains continuity of the alimentary tract, reducing dumping phenomena and preventing excessive incretin surges. Distal intestinal nutrient exposure enhances glucagon-like peptide-1 and peptide YY release, promoting satiety and improving insulin sensitivity without inducing the glycemic volatility associated with more proximal bypass [9, 18, 19]. The 300-cm common channel length reflects prior evidence indicating that this configuration provides balanced metabolic effect and nutritional safety, as demonstrated in Australian SADI-S cohorts including our group’s earlier work [10, 21, 22].

SADI-S also offers potential advantages over SG and biliopancreatic diversion with duodenal switch (BPD-DS). SG preserves pyloric function but exerts limited hormonal and bile-acid effects relative to diversionary procedures, resulting in more modest metabolic change [29, 30]. OAGB provides strong metabolic benefits but carries a recognized risk of bile reflux [31]. BPD-DS produces powerful metabolic effects but with substantially greater malabsorptive burden and nutritional instability [32–34]. Collectively, these observations suggest that SADI-S offers a favorable balance between metabolic potency and physiological stability for adults with T1DM. Mechanistic and comparative studies evaluating metabolic surgery, glycemic variability, nutritional outcomes, and quality-of-life measures provide important context for interpreting long-term outcomes following SADI-S in this cohort [27, 29, 31, 32, 35].

Nutritional safety is a critical consideration in insulin-dependent patients, for whom stable macronutrient absorption and micronutrient status influence insulin dosing. In this cohort, no patient developed severe micronutrient deficiency or protein–calorie malnutrition. Transient deficiencies in vitamins A, D, and folate were detected and corrected with dietary guidance and supplementation. These findings align with previous reports demonstrating favorable long-term nutritional outcomes with SADI-S using a 300-cm common channel, including our four-year Australian experience [21].

Two patients presented with EuDKA early in the series, both occurring within one week of surgery when a day-1 discharge protocol was used. Following protocol revision to extend inpatient monitoring to postoperative day three, no further EuDKA occurred. These events highlight the vulnerability of T1DM patients during periods of restricted oral intake and relative insulin under-dosing, reinforcing the importance of structured postoperative insulin protocols, ketone surveillance, and early carbohydrate administration [25, 26, 36].

Strengths of this study include the five-year duration of follow-up, use of CGM-based metrics, comprehensive nutritional monitoring, and inclusion of validated patient-reported outcome measures. The most important contribution is that it represents the first long-term evaluation of SADI-S in adults with T1DM, providing new clinical insight into metabolic, glycemic, and quality-of-life outcomes in this understudied population. Limitations include the small cohort size and incomplete long-term CGM availability. However, consistency across anthropometric, glycemic, nutritional, and patient-reported outcomes supports internal validity. Future research should involve multicenter longitudinal cohorts, include mechanistic studies examining incretin and bile-acid physiology in T1DM after SADI-S, and directly compare SADI-S with SG, RYGB, and OAGB using standardized CGM metrics including TIR, TAR, TBR, and CV.

Overall, SADI-S appears to offer a physiologically favorable, metabolically effective, and nutritionally safe option for selected adults with T1DM and obesity, demonstrating durable improvements in weight, insulin requirements, glycemic stability, and patient-reported outcomes over five years. Larger prospective studies are needed to confirm these findings and to clarify the role of SADI-S in the management of obesity and insulin resistance in T1DM.

## Data Availability

All data used in this study were fully de-identified prior to analysis. No identifiable personal information (including names, dates of birth, record numbers, addresses, or contact details) was collected, stored, or accessed by the investigators. Continuous glucose monitoring (CGM) metrics and patient-reported outcome measures were collected using secure, anonymised digital forms. All patients provided written consent for the use of their anonymised clinical information for research and publication.Data handling and storage complied with applicable national privacy regulations, institutional standards, and international best-practice guidelines. No identifiable or sensitive personal data is included in the manuscript or associated files.De-identified data supporting the findings of this study are available from the corresponding author upon reasonable request.
